# Evolutionary Evidence of Algal Polysaccharide Degradation Acquisition by *Pseudoalteromonas carrageenovora* 9^T^ to Adapt to Macroalgal Niches

**DOI:** 10.3389/fmicb.2018.02740

**Published:** 2018-11-22

**Authors:** Angélique Gobet, Tristan Barbeyron, Maria Matard-Mann, Ghislaine Magdelenat, David Vallenet, Eric Duchaud, Gurvan Michel

**Affiliations:** ^1^Sorbonne Université, CNRS, Integrative Biology of Marine Models (LBI2M), Station Biologique de Roscoff (SBR), Roscoff, France; ^2^Amadéite SAS, “Pôle Biotechnologique” du Haut du Bois, Bréhan, France; ^3^Génomique Métabolique, Genoscope, Institut François Jacob, CEA, CNRS, Université d’Evry, Université Paris-Saclay, Evry, France; ^4^VIM, INRA, Université Paris-Saclay, Jouy-en-Josas, France

**Keywords:** carrageenan, CAZymes, alginate, *Gammaproteobacteria*, *Pseudoalteromonas*, marine bacteria, algal holobiont, biofilm

## Abstract

About half of seaweed biomass is composed of polysaccharides. Most of these complex polymers have a marked polyanionic character. For instance, the red algal cell wall is mainly composed of sulfated galactans, agars and carrageenans, while brown algae contain alginate and fucose-containing sulfated polysaccharides (FCSP) as cell wall polysaccharides. Some marine heterotrophic bacteria have developed abilities to grow on such macroalgal polysaccharides. This is the case of *Pseudoalteromonas carrageenovora* 9^T^ (ATCC 43555^T^), a marine gammaproteobacterium isolated in 1955 and which was an early model organism for studying carrageenan catabolism. We present here the genomic analysis of *P. carrageenovora*. Its genome is composed of two chromosomes and of a large plasmid encompassing 109 protein-coding genes. *P. carrageenovora* possesses a diverse repertoire of carbohydrate-active enzymes (CAZymes), notably specific for the degradation of macroalgal polysaccharides (laminarin, alginate, FCSP, carrageenans). We confirm these predicted capacities by screening the growth of *P. carrageenovora* with a large collection of carbohydrates. Most of these CAZyme genes constitute clusters located either in the large chromosome or in the small one. Unexpectedly, all the carrageenan catabolism-related genes are found in the plasmid, suggesting that *P. carrageenovora* acquired its hallmark capacity for carrageenan degradation by horizontal gene transfer (HGT). Whereas *P. carrageenovora* is able to use lambda-carrageenan as a sole carbon source, genomic and physiological analyses demonstrate that its catabolic pathway for kappa- and iota-carrageenan is incomplete. This is due to the absence of the recently discovered 3,6-anhydro-D-galactosidase genes (GH127 and GH129 families). A genomic comparison with 52 *Pseudoalteromonas* strains confirms that carrageenan catabolism has been recently acquired only in a few species. Even though the loci for cellulose biosynthesis and alginate utilization are located on the chromosomes, they were also horizontally acquired. However, these HGTs occurred earlier in the evolution of the *Pseudoalteromonas* genus, the cellulose- and alginate-related loci being essentially present in one large, late-diverging clade (LDC). Altogether, the capacities to degrade cell wall polysaccharides from macroalgae are not ancestral in the *Pseudoalteromonas* genus. Such catabolism in *P. carrageenovora* resulted from a succession of HGTs, likely allowing an adaptation to the life on the macroalgal surface.

## Introduction

Marine macroalgae are complex multicellular photosynthetic organisms, evolutionary distinct from land plants, and constitute a large primary biomass in coastal ecosystems. Polysaccharides constitute about half of the algal tissues and can be found either as carbon storage or cell wall compounds (Kloareg and Quatrano, 1988). Like plant polysaccharides, macroalgal polysaccharides can be neutral (e.g., starch, laminarin, cellulose), or polyanionic (Ficko-Blean et al., 2015). Red algae (Rhodophyta) typically contain sulfated xylans or sulfated galactans (agars or carrageenans) in their cell wall (Deniaud et al., 2003). Among them, the main three types of carrageenans include kappa-, iota-, and lambda-carrageenan, chains of disaccharidic galactans which contain one, two or three sulfate groups, respectively (Michel et al., 2006). Brown algae (Phaeophyceae) produce laminarin as storage compound and contain fucose-containing sulfated polysaccharides (FCSP) and alginate in their cell wall (Popper et al., 2011; Deniaud-Bouët et al., 2017). Alginates account for 10–45% of the dry weight of brown algal cell wall. They are anionic polymers made of alpha-(1,4)-L-guluronate and beta-(1,4)-D-mannuronate aligned in blocks (Mabeau and Kloareg, 1987; Kloareg and Quatrano, 1988; Deniaud-Bouët et al., 2014). These different types of polysaccharides represent a large choice of carbon sources for marine heterotrophic bacteria (MHB, Egan et al., 2013; Martin et al., 2014). To utilize such carbon sources, MHB produce specific carbohydrate-active enzymes (CAZymes), classified as glycoside hydrolases (GH), polysaccharide lyases (PL) or other types of modifying enzymes (Lombard et al., 2014) and sulfatases (Barbeyron et al., 2016a). Indeed, several members of the *Bacteroidetes*, *Planctomycetes* or *Gammaproteobacteria*, secrete sulfatases, agarases or carrageenases to degrade red macroalgal galactans or present alginolytic systems allowing the degradation of brown algal polysaccharides (Michel et al., 2006; Thomas et al., 2012; Wegner et al., 2013; Martin et al., 2014; Neumann et al., 2015). In *Bacteroidetes*, gene clusters are described as polysaccharide utilization loci (PUL) including CAZymes, recognition proteins, transporters and coregulated genes that act together for the detection and uptake of a specific polysaccharide (Bjursell et al., 2006). The specificity of PULs unique to *Bacteroidetes* is that they include a pair of *susC* and *susD* homologs. These genes, respectively, encode a TonB-dependent receptor (TBDR), localized in the outer membrane of gram-negative bacteria and known to actively transport large molecules to the periplasmic space (Noinaj et al., 2010), and a carbohydrate-binding lipoprotein (Martens et al., 2009). Similar carbohydrate utilization loci are also found in other bacterial phyla, usually including a standalone TBDR gene but without *susC*-like gene. Such gene clusters have been recently proposed to be also named PULs (Ficko-Blean et al., 2017; Grondin et al., 2017).

Among the *Gammaproteobacteria*, the *Pseudoalteromonas* genus represents exclusively MHB and they are usually producers of biologically active extracellular agents and active producer of biofilms (Bowman, 2007). They can secrete anti-microbial or anti-viral compounds, display anti-fouling activities or produce macroalgal polysaccharide-degrading enzymes. They are frequently associated with marine eukaryotic hosts such as fishes, mussels, sponges or macroalgae (Holmström and Kjelleberg, 1999; Martin et al., 2014). For instance, the genomic analysis of *Pseudoalteromonas tunicata*, isolated from the green macroalga *Ulva lactuca* and the tunicate *Ciona intestinalis*, showed its ability to attach to marine surfaces, to form biofilms and to produce bioactive compounds (Thomas et al., 2008). *Pseudoalteromonas* species can also degrade complex polysaccharides and such capacities can be acquired by horizontal gene transfers (HGT) as recently shown for the pectin degradation pathway in *P. haloplanktis* ANT/505 (Hehemann et al., 2017). Our study will focus on *Pseudoalteromonas carrageenovora* 9^T^, which was isolated among a pool of bacteria from marine waters and algae around Nova Scotia (Yaphe and Baxter, 1955) and was the first microorganism shown to produce carrageenases and carrageenan-specific sulfatases (McLean and Williamson, 1979a,b, 1981). The GH16 family kappa-carrageenase from *P. carrageenovora* was the first to be genetically (Barbeyron et al., 1994), biochemically (Potin et al., 1995), and structurally characterized (Michel et al., 1999, 2001). Its lambda-carrageenase was also purified and characterized and the corresponding gene *cglA* was cloned (Johnston and McCandless, 1973; Guibet et al., 2007), defining the GH150 family (Lombard et al., 2014). *P. carrageenovora* is also able to degrade fucoidin (former name for FCSP) and alginate from the brown alga *Fucus vesiculosus* (Yaphe and Morgan, 1959; Akagawa-Matsushita et al., 1992). Furthermore, an arylsulfatase was cloned from this gammaproteobacterium (Barbeyron et al., 1995). This protein is the first characterized member of the S4 family of sulfatases, which belong to the zinc-dependent beta-lactamase superfamily (Barbeyron et al., 2016a). All these activities likely reflect that the bacterium *P. carrageenovora* has a macroalgae-associated lifestyle. To further investigate to which extent this bacterium is adapted to such lifestyle, we have explored the carbohydrate metabolism in *P. carrageenovora*’s genome and validated the predicted activities by profiling bacterial growth on selected carbon sources. Our work has also brought new insights on the following evolutionary and ecological questions: (*i*) are macroalgal polysaccharide-degrading genes commonly found in *Pseudoalteromonas* genomes? (*ii*) did *P. carrageenovora* acquire such activities by HGT from other MHB? (*iii*) are there genetic traits suggesting an adaptation of *P. carrageenovora* to an algae-associated lifestyle?

## Materials and Methods

### Culture Conditions and Catabolic Profiling of *P. carrageenovora* 9^T^

For all subsequent experiments, pre-cultures of *P. carrageenovora* were prepared in 5 mL ZoBell medium 2216E (5 g.L^-1^ tryptone, 1 g.L^-1^ yeast extract, sea water; (ZoBell, 1941) at 20°C and under an agitation of 400 rpm, until reaching the maximum growth phase (OD_600_ of about 2). Bacterial growth of *P. carrageenovora* was screened using a carbohydrate collection as followed. Triplicated cultures were prepared in sterile 48-well plates on a medium consisting of 200 μL of a marine mineral medium (MMM) (for one liter of medium: 24.7 g NaCl, 6.3 g MgSO_4_.7H_2_O, 4.6 g MgCl_2_.H_2_O, 0.7 g KCl, 20 mg FeSO_4_.7H_2_O, 200 mg NaHCO_3_, 0.6 g CaCl_2_, 100 mg K_2_HPO_4_, a mix of 11 vitamins (5 mg pyridoxine HCl, 1 mg nicotinic acid, 1 mg thiamine HCl, 1 mg riboflavin, 1 mg D,L-pantothenic acid, 10 mg 4-aminobenzoic acid, 1 mg D-biotin, 1 mg folic acid, 1 mg cyanocobalamine, 5 mg orotic acid, 1 mg ascorbic acid), Tris-HCl 50 mM pH 8, and 2 g NH_4_Cl) and 800 μL of one of the 44 oligo- or poly-saccharides (Supplementary Table S1) with a final concentration of 4 g.L^-1^ (Thomas et al., 2011). Cells from the 5 mL pre-culture of *P. carrageenovora* were washed twice in the same volume of artificial seawater (for one liter of water: 24.7 g NaCl, 6.3 g MgSO_4_.7H_2_O, 4.6 g MgCl_2_.H_2_O, 0.7 g KCl) after centrifugation for 10 min at 4,000 *g* at room temperature. This inoculum was added to each well for a final OD_600_ of about 0.05 and cultures were grown at 20°C at 400 rpm. The growth of *P. carrageenovora* with each carbon source was measured after 1 week at OD_600_ using the Spark^®^ Multimode Microplate reader (Tecan).

### Genomic DNA Extraction

Material for genomic DNA extraction was prepared by transferring 1 mL of the pre-culture of *P. carrageenovora* in 200 mL ZoBell medium 2216E at 20°C, with shaking at 200 rpm, until reaching the maximum growth phase. The culture was then centrifuged at 3,000 *g* for 15 min and the resulting pellet was stored at -20°C until further processing. Genomic DNA from *P. carrageenovora* was extracted following the method from Barbeyron et al. (1984) with some modifications. Five to ten grams of cells were suspended in 25 mL of 50 mM Tris-HCl (pH 8)-25% sucrose. A volume of 5 mL of lysis buffer (50 mM Tris-HCl (pH 8), 5 mM EDTA, 50 mM NaCl) and 50 mg of lysozyme were then added and the resulting mix was incubated for 15 min at room temperature to form spheroplasts. After the addition of 6 mL of 100 mM EDTA (pH 8), lysis started during the incubation of 10 min on ice and finished after the addition of 1.5 mL of 2% SDS and 25 mL of lysis solution (50 mM Tris-HCl (pH 8), 100 mM EDTA, 100 mM NaCl). Proteins were hydrolyzed with 40 mg of proteinase K and the mix was incubated for 1 h at 50°C. Sodium perchlorate (1 M) was added to break DNA-proteins bonds. Nucleic acids were then purified following a phenol/chloroform extraction. The aqueous phase containing the nucleic acids was recuperated and the remaining proteins were removed by a chloroform/isoamyl alcohol 24:1 extraction. Nucleic acids were precipitated with 0.6 volume of isopropanol on a glass stick, washed in 70% EtOH, dehydrated in 99% EtOH and dried overnight. Nucleic acids were then resuspended in 20–30 mL of a solution of 10 mM Tris-HCl (pH 8) and 1 mM EDTA and kept at 4°C until further processing.

### Genome Sequencing and Genome Assembly

*P. carrageenovora* 9^T^ genome was sequenced using a combination of Sanger (ABI3730, Applied Biosystems) and Solexa (GAIIx, 2 × 74 paired-end reads with 300 bp insert size) reads. The 19,117,159 GAIIx-filtered sequence (estimated coverage of 300×) reads were assembled in 51 contigs (>1 kb) using Velvet (Zerbino and Birney, 2008). Contigs were ordered using an optical map (Argus system, OpGene, MD, United States) and the *SpeI* restriction enzyme (Latreille et al., 2007). All gaps (except three in the large chromosome) were filled using PCR sequencing. The Velvet contigs and the Sanger reads were assembled using the whole-genome shotgun assembler Phrap and the assembly was visualized with the interface Consed (Ewing et al., 2005). The final assembly encompassing two chromosomes and a plasmid was validated using the optical map.

### Genome Annotation

Automatic functional annotation of all genes was done through the MicroScope platform (Vallenet et al., 2017). CAZymes were specifically identified by homology (at least 30% sequence identity on 80% sequence length) with characterized enzymes selected in each family of the CAZY database (Lombard et al., 2014)^1^. Annotations were then cross-validated using the BlastP alignments against the UniProtKB/SwissProt database (The UniProt Consortium, 2014) and Pfam domain predictions (Punta et al., 2012)^2^. Each sequence was assigned to a CAZyme family and, when it was possible, to an EC number. Sulfatase genes were assigned to their corresponding SulfAtlas (sub)family (Barbeyron et al., 2016a)^3^. Manual annotation of key genes was done using the MicroScope platform and the ngKLAST software (Nguyen and Lavenier, 2009).

### Phylogeny of 16S rRNA Gene and Other Selected Genes

The phylogenetic position of *P. carrageenovora* 9^T^, was determined among 87 other *Pseudoalteromonas* strains (52 strains with a publicly available genome and 35 taxonomically characterized strains) and selected representative sequences of other genera of the *Alteromonadales* order. The 16S rRNA sequences from 409 species of *Alteromonadales* were aligned using MAFFT v.7 with the L-INS-i algorithm (Katoh and Standley, 2013). The multiple sequence alignment was visualized using the Jalview software v.11.0 (Clamp et al., 2004) and non-aligned regions were removed. A total of 1,423 positions were used for the phylogeny. Phylogeny was made using MEGA v.5.1 (Tamura et al., 2011). The evolutionary history was inferred using Neighbor-Joining method (Saitou and Nei, 1987) and the evolutionary distances were computed using the Kimura-two-parameters as evolutive model (Kimura, 1980). The gaps-containing sites were treated using the Pairwise-deletion option. The reliability of the trees was tested by bootstrap analysis using 1,000 resamplings of the dataset (Felsenstein, 2009).

### Data Availability

Annotated genome sequences were deposited in EMBL, accession number GCA_900239935.

## Results

### General Carbohydrate Degrading and Biosynthesis Capacities of *P. carrageenovora*

The genome of *P. carrageenovora* is composed of a larger chromosome of 3,620 kb and 3,183 coding DNA sequences (CDS, chromosome I), a smaller chromosome of 820 kb and 708 CDS (chromosome II), and a plasmid of 143 kb and 109 CDS. Each replicon has an average GC content of 39.55, 39.13, and 37.74%, respectively (Table 1). The genome encodes several CAZymes, including 47 GH and 5 PL that may degrade endogenous and exogenous carbohydrates, and 31 glycosyltransferases (GT) for carbohydrate biosynthesis, which correspond to 22 GH families, 4 PL families and 13 GT families, respectively (Supplementary Table S2). The genome also encodes 10 sulfatases, 9 belonging to four S1 subfamilies and one to the S4 family, and they may participate in the degradation of sulfated carbohydrates (Barbeyron et al., 2016a). The CAZyme repertoire includes functions of the carbohydrate metabolism regularly found in *Bacteria* such as the ability to synthesize and recycle its own peptidoglycan and its own glycogen. However, most CAZymes and sulfatases display signal peptides and are predicted to be periplasmic, membrane-anchored or secreted, suggesting that these enzymes target external carbohydrate substrates. Some of the CAZyme and sulfatase genes are located close to putative TBDR (oligosaccharide import into the periplasm), major facility superfamily (MFS)-transporter or sodium/solute symporters (SSS, cytoplasm uptake of monosaccharide or small sugars), and transcriptional regulators, and thus are part of PULs. Eleven of the 58 TBDR genes found in the genome belong to nine PULs likely specific for macroalgal polysaccharides for instance, alginate, the brown algal storage polysaccharide laminarin, or various carrageenans (Supplementary Table S3).

**Table 1:** General statistics of the *Pseudoalteromonas carrageenovora* 9^T^ genome.

		Chromosome I	Chromosome II	Plasmid
	Size (bp)	3,620,649	820,350	143,981
	GC %	39.55	39.13	37.74
	Total number of CDS	3,135	694	102
	Average CDS length (bp)	1000.3	1010.2	1114.79
	% CDS	87.53	86.72	84.2
	Number of rRNA operons	7	1	0
	Number of tRNAs	97	0	0
**CAZymes and sulfatases**	Total number of GH sequences (# families)	25 (15)	10 (7)	12 (6)
	Ratio # GH sequences/# CDS	0.79%	1.44%	11.76%
	Total number of GT sequences (# families)	24 (9)	7 (7)	0
	Ratio # GT sequences/# CDS	0.76%	1.01%	0
	Total number of PL sequences (# families)	5 (4)	0	0
	Ratio # PL sequences/# CDS	0.16%	0	0
	Total number of sulfatase sequences (# families)	2 (1)	0	8 (4)
	Ratio # Sulfatase sequences/# CDS	0.06%	0	7.84%


**CDS, Coding DNA Sequence. GH, glycoside hydrolases; GT, glycosyltransferases; PL, polysaccharide lyases.**

To validate these activities, growth of *P. carrageenovora* was monitored on a marine mineral minimum medium (MMM) containing one of 44 carbohydrate sources (Supplementary Table S1). They include mono- and di-saccharides, as well as polysaccharides that can either be found in algal cell wall or storage (e.g., agar, porphyran, several types of carrageenans, alginate, laminarin), in vascular plants (e.g., cellulose, xylan, xyloglucan) or in animal tissues (e.g., chondroitin sulfate A and C, hyaluronic acid). *P. carrageenovora* could grow on two of the four monosaccharides tested, three of the four disaccharides and on 11 of the other 36 polysaccharides. The bacterium was able to grow on storage polysaccharides from algae such as starch (growth on amylopectin) and brown algal laminarin as well as on cell wall polysaccharides from brown algae such as alginate and fucoidan (FCSP from *Pelvetia caniculata* and *Ascophyllum nodosum*), and from red algae such as lambda-carrageenan (Table 2). The bacterium was not able to grow on polysaccharides unique to land plants or animals, suggesting a specificity for macroalgal degradation.

**Table 2:** Carbohydrate utilization by *Pseudoalteromonas carrageenovora* 9^T^.

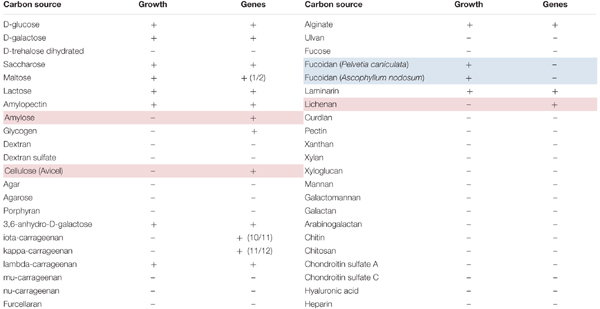

*Growth of the bacterium was tested on each of the 44 carbon sources using three biological replicates. Observed growth is indicated by (+) while no growth observed is indicated by (–). The presence of the necessary genes to use the corresponding carbon source is indicated by (+) and an absence of the necessary genes by (–) this is based on amino-acid sequence identity with genes present in metabolic pathways characterized in other organisms (for further details see Supplementary Table S4). Substrates are highlighted in blue when the substrate is assimilated but no corresponding known gene could be found and in orange when the substrate is not assimilated but the corresponding genes are present in the genome.*

We screened the genome for the genes needed to use each carbon source. In most cases, the utilization of a given carbon source could be explained by the presence of all the necessary genes (Table 2 and Supplementary Table S4). In the cases of the two brown algal FCSPs, the bacterium grew but the genome contains no GH107 fucoidanase gene (Colin et al., 2006). However, the knowledge on FCSP biodegradation is currently very limited (Deniaud-Bouët et al., 2017) and one can expect that novel, uncharacterized CAZyme families are involved in this catabolism. In other cases, genes homologous to characterized CAZymes were present in the genome but the bacterium did not grow on the corresponding substrate. In the case of amylose, this substrate is not soluble in water as its helical structure is tightly packed, making it not as accessible to the bacterium as it is under the amylopectin form. Despite the presence of a potentially secreted cellulase in the genome (GH5_2-CBM5 family, PCAR9_b0611), the bacterium did not grow on crystalline cellulose from plants. However, the GH5 family is highly polyspecific (Aspeborg et al., 2012) and this GH5 enzyme most likely acts on other substrates such as hemicellulose. Despite the presence of three GH16 beta-glucanases, there was also no growth on mixed-linked glucan (lichenan, beta-1,3-1,4-glucan). Still, *P. carrageenovora* is able to use laminarin (beta-1,3-glucan), suggesting that these GH16 enzymes are strict laminarinases. Other explanations for such discrepancies between growth and genomic content may be that the bacterium is not able to detect or uptake these polysaccharides, or that genes corresponding to polysaccharide degradation are not expressed in the MMM conditions.

### Carrageenan Catabolism in *P. carrageenovora*

#### Carrageenan-Specific Genes Are Located on the Plasmid

**FIGURE 1:**
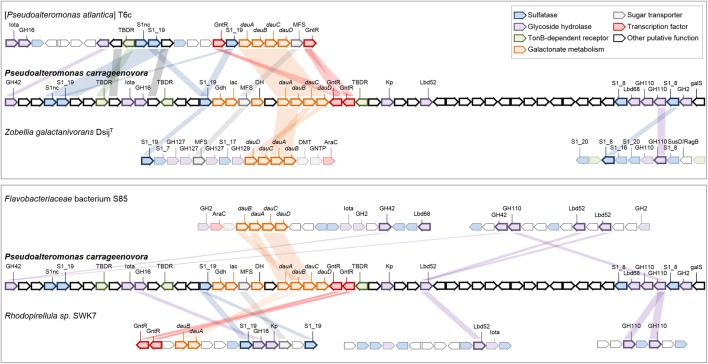
Genes of PUL 7 involved in the catabolism of carrageenan in the genome of *P. carrageenovora* 9^T^ and orthologous genes from other MHB taxa. Each arrow indicates a separate gene. Orthologous genes in the genome of [*Pseudoalteromonas atlantica*] T6c (gene locus ID: Patl_0879 to Patl_0901), the *Planctomycetes Rhodopirellula* sp. SWK7 (gene locus ID: ANOQ_1350023 to ANOQ_1350036 on the left, ANOQ_120161 to ANOQ_120173 in the middle, and ANOQ_790111 to ANOQ_790117 on the right) and the *Bacteroidetes Zobellia galactanivorans* Dsij^T^ (gene locus ID: ZGAL_3145 to ZGAL_3159 on the left and ZGAL_2194 to ZGAL_2203 on the right) and *Flavobacteriaceae* bacterium S85 (gene locus ID: AFPK_670040 to AFPK_670057 on the left and AFPK_440002 to AFPK_440015 on the right) are depicted by hues colored according to their putative function (further details in Supplementary Table S8). When available, the family of the gene is indicated above the arrow. Kp, Kappa-carrageenase; Iota, iota-carrageenase; Gdh, Galactonate dehydratase; lac, putative sugar lactone lactonase; DH, short-chain dehydrogenase/reductase; *dauA*, 3,6-anhydro-D-galactose dehydrogenase; *dauB*, 3,6-anhydro-D-galactonate cycloisomerase; *dauC*, 2-keto-3-deoxy-D-galactonate kinase; *dauD*, 2-keto-3-deoxy-D-galactonate aldolase; GntR, Galactonate operon transcriptional repressor; Lbd52 and Lbd68, Pre-lambda-carrageenases; galS, HTH-type transcriptional regulator. The bacterium strain [*Pseudoalteromonas atlantica*] T6c probably belongs to the genus *Paraglaciecola*, has shown by a 100% sequence similarity with the hit of accession number CP000388 using Blast search on EZBioCloud.

CAZymes and sulfatases from *P. carrageenovora*’s plasmid are almost exclusively involved in the catabolism of carrageenans and represent large proportions of the CDS content, with 11% GH and 7.34% sulfatases (Table 1). Indeed, the genes of the characterized kappa-carrageenase *Pc*CgkA (Michel et al., 2001) and lambda-carrageenase *Pc*CglA (Guibet et al., 2007) are located on the plasmid (PCAR9_p0048 and PCAR9_p0052, respectively). There are also plasmid-encoded proteins homologous to carrageenan-specific enzymes characterized in other marine gammaproteobacteria [PCAR9_p0019 and PCAR9_p0029: 61 and 76% sequence identity with the exo-beta-carrageenase (GH42-like) and the endo-beta-carrageenase (GH16) from *Paraglaciecola hydrolytica* S66, (Schultz-Johansen et al., 2018); PCAR9_p0028: 95% with the GH82 iota-carrageenase from *Pseudoalteromonas* sp. An33 (Martin et al., 2016)] or from the flavobacterium *Z. galactactanivorans* Dsij (Ficko-Blean et al., 2017): two iota-carrageenan G4S-sulfatases (PCAR9_p0023 and PCAR9_p0034, 53 and 40% with *Zg*CgsA, respectively), 3,6-anhydro-D-galactose dehydrogenase (PCAR9_p0040, 48% with *Zg*DauA), 3,6-anhydro-D-galactonate cycloisomerase (PCAR9_p0041, 40% with *Zg*DauB), 2-keto-3-deoxy-D-galactonate kinase (PCAR9_p0042, 32% with *Zg*DauC) and 2-keto-3-deoxy-D-galactonate aldolase (PCAR9_p0042, 39% with *Zg*DauD). All these genes are located in a large PUL encompassing 33 genes (PUL 7, Figure 1 and Supplementary Table S5). This PUL also contains a third S1 sulfatase (PCAR9_p0022, S1_NC) which likely desulfates carrageenan although its regioselectivity is uncertain, three TBDR genes (PCAR9_p0026, PCAR9_p0031, PCAR9_p0046) and an MFS-transporter (PCAR9_p0037) which are potentially responsible for the uptake of oligo-carrageenans and carrageenan-derived monosaccharides.

**FIGURE 2:**
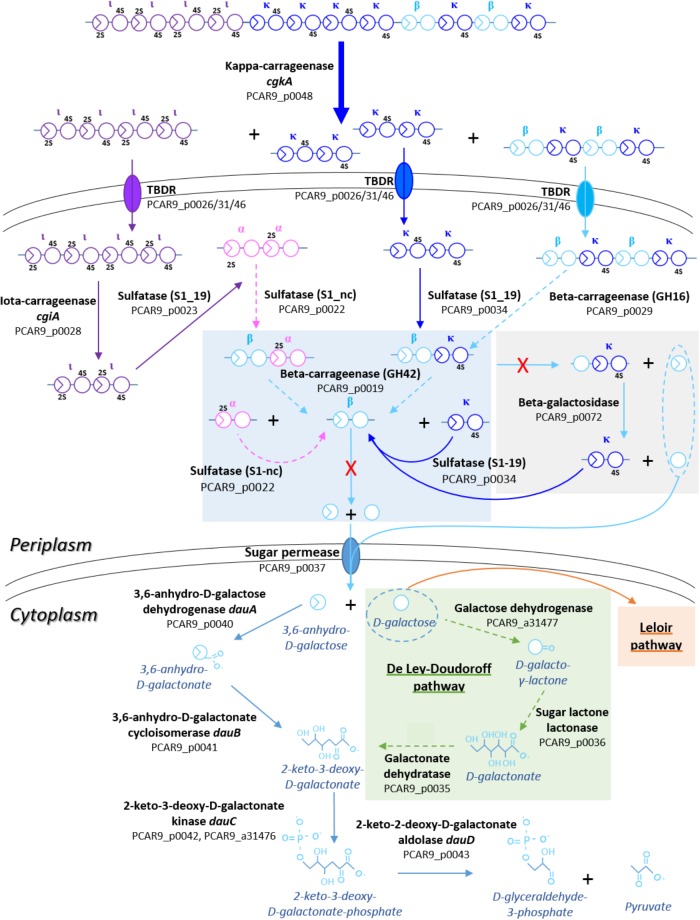
Theoretical representation of degradation pathways of kappa family carrageenans by enzymes encoded in the carrageenolytic plasmid of *P. carrageenovora*. Arrows in bold indicate enzymatic activities experimentally demonstrated, simple arrows indicate activities predicted by sequence homology with enzymes characterized in other organisms; dashed arrows indicate putative activities. Genes associated with enzymatic activities are indicated in brackets, in bold if experimentally checked or followed by a question mark if hypothetical. Steps specific to each carrageenan are colored in purple for iota-/alpha- and in blue for kappa-/iota-carrageenan. A red cross indicates the missing GH127/129 in the pathway.

Altogether, *P. carrageenovora* is well equipped for the degradation of kappa family carrageenans (with its kappa-, iota-, and beta-carrageenases and some carrageenan sulfatases) and for the import of these degradation products (Figure 2). The presence of the *dauABCD* genes suggests that this bacterium has the capacity to convert released 3,6-anhydro-D-galactose moieties into pyruvate and glyceraldehyde-3-P (Ficko-Blean et al., 2017). The bacterium is also able to use D-galactose utilization as the Leloir pathway (Leloir, 1951) is complete on chromosome I (aldose-1-epimerase, PCAR9_a31484; galactokinase, PCAR9_a31471; galactose-1-phosphate uridylyltransferase, PCAR9_a31470, and UDP-galactose-4-epimerase, PCAR9_a31469), suggesting that this catabolic route is ancestral to the acquisition of the plasmid. Interestingly, PUL 7 also includes two genes potentially involved in the De Ley-Doudoroff pathway (D-galactonate dehydratase, PCAR9_p0035; sugar lactone lactonase PCAR9_p0036). Two neighboring genes from chromosome I (D-galactose dehydrogenase, PCAR9_a31477; 2-dehydro-3-deoxygalactonokinase, PCAR9_a31476) might complete this alternative route for the D-galactose utilization (De Ley and Doudoroff, 1957). However, *P. carrageenovora* seems to lack 3,6-anhydro-D-galactosidases. These enzymes are found in the GH127 and GH129 families and are essential for releasing 3,6-anhydro-D-galactoses from oligo-carrageenans (Ficko-Blean et al., 2017). Surprisingly, GH127 and GH129 genes are found neither on the plasmid nor on the chromosomes, suggesting that *P. carrageenovora* is unable to release 3,6-anhydro-D-galactose from carrageenan degradation products and thus unable to use kappa-family carrageenans alone (Figure 2).

Two other PULs are located on the plasmid. PUL 8 (PCAR9_p0067-PCAR9_p0073) includes a second GH150 lambda-carrageenase, surrounded by a GH2 beta-galactosidase, two GH110 enzymes and two S1_8 sulfatases (Figure 1 and Supplementary Table S5). Interestingly, the GH110 family currently contains 1,3-alpha-galactosidases from gut *Bacteroides* which catalyze the removal of alpha-1,3-linked galactose residues of blood group B antigens (Liu et al., 2007). This suggests that *P. carrageenovora* GH110 enzymes are also 1,3-alpha-galactosidases, but most likely acting on oligo-lambda-carrageenans released by GH150 lambda-carrageenases (which cleave beta-1,4 linkages). The PUL 8 GH2 beta-galactosidase likely acts in synergy with the GH110 enzymes to completely hydrolyze oligo-lambda-carrageenans into D-galactose, similarly, to the interplay between GH127/GH129 and GH2 which alternatively cleave alpha-1,3 and beta-1,4 linkages in kappa family oligo-carrageenans (Ficko-Blean et al., 2017). Finally, the third PUL (PUL 9, PCAR9_p0107-PCAR9_p0113) includes a family S1_8 sulfatase, two family S1_15 sulfatases, a galactokinase, a TBDR and a SSS transporter genes. The presence of an S1_8 sulfatase (also found in PUL 8) and of a galactokinase suggests that PUL 9 is involved in carrageenan catabolism, although it is less certain than in the cases of PULs 7 and 8.

#### Physiological Analyses of Carrageenan Utilization

**FIGURE 3:**
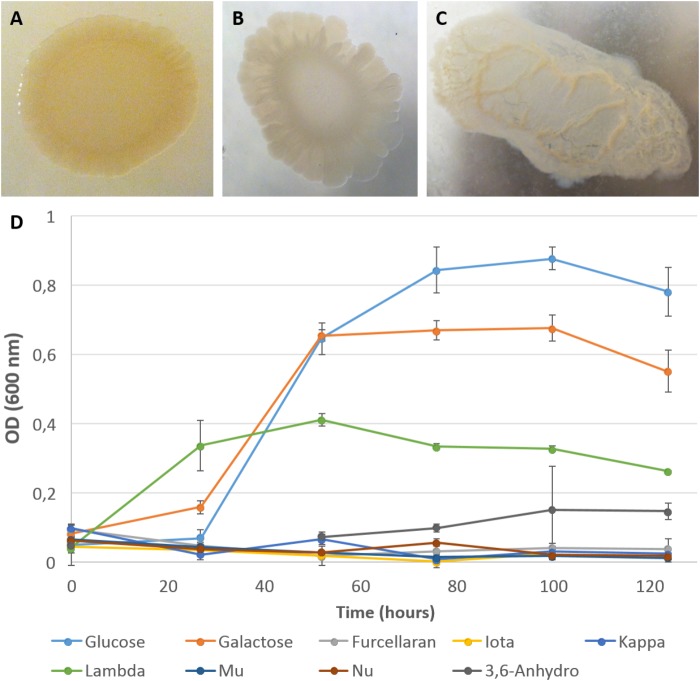
Growth of *P. carrageenovora* on a selection of galactans. Colonies of *P. carrageenovora* on ZoBell medium 2216E gelose plates containing 1.5% agar **(A)**, 2% iota-carrageenan **(B)**, or 1% kappa-carrageenan **(C)**, 13 days after inoculation. Colonies on iota- and kappa-carrageenan plates showed a depression and liquefaction in the respective media. Kinetic of *P. carrageenovora* growth was monitored by measuring optical density (OD_600_) on a minimum mineral medium with either one of the nine carbohydrate sources tested. Curves indicate the mean optical density measured for the biological triplicates and bars indicate the standard deviation **(D)**. Glucose, D-glucose; Galactose, D-galactose; Iota, iota-carrageenan; Kappa, kappa-carrageenan; Lambda, lambda-carrageenan; Mu, mu-carrageenan; Nu, nu-carrageenan; 3,6-Anhydro, 3,6-anhydro-D-galactose.

In parallel to the genomic analyses, growth experiments were undertaken using two types of medium: (*i*) a ZoBell medium gelified with agar, kappa- or iota-carrageenans (Figures 3A–C), and (*ii*) a liquid MMM complemented by D-galactose, 3,6-anhydro-D-galactose or a collection of carrageenans (Figure 3D). Growth experiments on gelified rich medium confirmed the ability of *P. carrageenovora* to hydrolyze kappa- and iota- carrageenans. Its capacity to degrade kappa- and lambda-carrageenans is well known in the literature (Yaphe and Baxter, 1955; Michel et al., 2001; Guibet et al., 2007), but it is the first time that *P. carrageenovora* is shown to be an iota-carrageenolytic bacterium. This suggests that the GH82 enzyme PCAR9_p0028 is indeed an expressed and active iota-carrageenase. In MMM, bacterial growth was observed on D-galactose as well as on 3,6-anhydro-D-galactose, consistently with the presence of the Leloir pathway and of the *dauABCD* genes in the genome. However, *P. carrageenovora* was unable to grow with kappa family carrageenans (i.e., kappa-, iota-, and beta-carrageenans) as sole carbon sources, confirming that these catabolic pathways are incomplete, most likely due to the absence of GH127 and GH129 genes in the genome. In contrast, *P. carrageenovora* grew well with lambda-carrageenan, confirming that this bacterium possesses all the enzymes necessary for using this sulfated galactan which has the particularity to lack 3,6-anhydro-D-galactose in its structure. It also validates that lambda-carrageenan catabolic pathway is separate from the iota-/kappa-carrageenan one.

#### Conservation of the Carrageenan Catabolisms in the *Pseudoalteromonas* Genus

To determine whether the carrageenolytic capacities of *P. carrageenovora* are common to the genus *Pseudoalteromonas*, we searched for homologs from PULs 7 and 8 in 52 *Pseudoalteromonas* genomes. Only six genomes belonging to the same late-diverging clade (LDC) in the 16S rRNA gene-based phylogeny presented the key genes of PUL 7, suggesting a recent acquisition of this PUL in these strains (Supplementary Figure S1 and Supplementary Tables S6, S7). The GH82 iota-carrageenase gene is absent in three of these genomes. Additionally, the GH150 lambda-carrageenase and GH110 genes (PUL 8) are absent from all *Pseudoalteromonas* genomes, suggesting their recent acquisition following that of the plasmid in *P. carrageenovora*. To determine the potential origin of PUL 7 and 8 genes, we searched for homologs in genomes of macroalgae-associated bacteria (Figure 1 and Supplementary Table S8). The carrageenan PUL organization in [*Pseudoalteromonas atlantica*] T6c (which is in fact a *Paraglaciecola* strain based on 16S rRNA gene phylogeny) was the most similar to that of PUL 7. The synteny of the four *dauABCD* genes seems to be kept between MHB within a same class (*Gammaproteobacteria* and *Flavobacteriia*). However, as in PUL 7, the *dauABCD* genes of *Flavobacteriaceae* bacterium S85 are in the vicinity of a GH82 iota-carrageenase gene while this is not the case in *Z. galactanivorans* Dsij^T^, suggesting that the genome of these flavobacteria were subjected to different gene rearrangements. The genome of the *Planctomycetes Rhodopirellula* sp. SWK7 contains some homologs from PUL 7 that show a different arrangement. For instance, only two of the four *dau* genes (*dauAB*) are present, suggesting gene loss in the corresponding PUL. These comparative genomics of *P. carrageenovora* and other MHB indicated that homologs from PUL 7 were arranged most differently in species belonging to different bacterial classes. Regarding PUL 8, we have identified a similar PUL in the marine *Flavobacteriaceae* bacterium S85, which also contains two GH150 lambda-carrageenases next to GH2 and GH110 genes. Another PUL containing also a GH150 lambda-carrageenase and GH110 genes is found in *Rhodopirellula* sp. SWK7 (Figure 1). This conservation of GH110 genes alongside lambda-carrageenase genes strengthen the hypothesis that these GH110 enzymes are 1,3-alpha-galactosidases specific for oligo-lambda-carrageenans. Interestingly, the PUL 23 of *Z. galactanivorans* Dsij (Barbeyron et al., 2016b) includes GH110 and S1_8 sulfatases highly similar to those of the lambda-carrageenan-specific PUL from *P. carrageenovora* and *Flavobacteriaceae* bacterium sp. S85. Although its genome is lacking GH150 gene, *Z. galactanivorans* Dsij is able to use lambda-carrageenan as a sole carbon source. This suggests that GH110s and S1_8 sulfatases from *Z. galactanivorans* Dsij are most likely specific for lambda-carrageenan and act in synergy with a lambda-carrageenase belonging to a novel, unidentified GH family.

### The Chromosomes Contain Carbohydrate-Related Genes Linked to the Adaptation to an Algae-Associated Ecological Niche

CAZymes and sulfatases are distributed differently between the chromosomes. Chromosome I contains enzymes involved in the catabolism of polysaccharides with 0.79% of the total number of CDS being GH, 0.76% CDS being PL and 0.06% CDS being sulfatases, and in the biosynthesis of polysaccharides with 0.76% CDS being GT. Chromosome II contains 1.42% CDS are GH and 0.85% CDS are GT but no PL nor sulfatases (Table 1 and Supplementary Table S2). These CAZymes and sulfatases include functions conserved in *Bacteria* (e.g., peptidoglycan or glycogen recycling) and functions rather specific to the genus *Pseudoalteromonas* while others may result from HGT events. CAZymes and sulfatases of the chromosomes are mostly involved in algal polysaccharide degradation and life in the biofilm.

#### Alginate Catabolism

**FIGURE 4:**
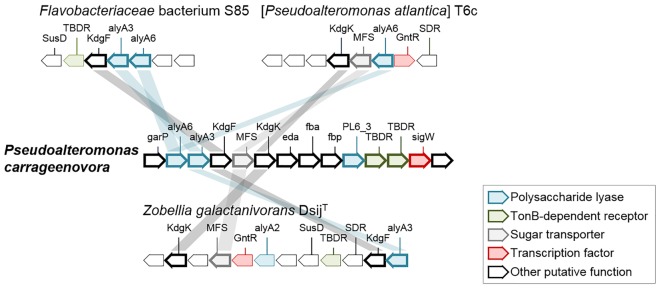
Genes of PUL 1 comprising enzymes involved in the catabolism of alginate in the genome of *P. carrageenovora*. Each arrow indicates a separate gene. Orthologous genes in the genome of [*Pseudoalteromonas atlantica*] T6c (gene locus ID: Patl_3654 to Patl_3661), the *Bacteroidetes Zobellia galactanivorans* Dsij^T^ (gene locus ID: ZGAL_2613 to ZGAL_2624) and *Flavobacteriaceac* bacterium S85 (gene locus ID: AFPK_720026 to AFPK_720032) are depicted by hues colored according to their putative function. When available, the family of the gene is indicated above the arrow. PL6, PL7, PL17_2, polysaccharide lyases from family 6, family 7, family 17_2; *garR*, 2-hydroxy-3-oxopropionate reductase; *KdgF*, Pectin degradation protein; *KdgK*, 2-dehydro-3-deoxygluconokinase; *eda*, KHG/KDPG aldolase; *fba*, Fructose-biphosphate aldolase; *fbp*, Fructose-1,6-biphosphatase class 1 2; MFS, Major Facilitator Superfamily; *sigW*, *GntR*, Sigma/transcriptional factor; SDR, short-chain dehydrogenase/reductase.

Algal polysaccharide catabolism is mostly localized on chromosome I. It encodes five secreted alginate lyases belonging to four polysaccharide lyase families (families PL6, PL7, PL17, and PL18). Three of these alginate lyase genes belong to a 14-gene cluster resembling the alginate utilization system characterized in *Z. galactanivorans* Dsij (Thomas et al., 2012). This cluster is named here PUL 1 (Figure 4 and Supplementary Table S3). There are two potential endo-alginate lyases from PL6 family (PCAR9_a20870, PCAR9_a20878) and one exo-alginate lyase from PL17 family (PCAR9_ a20871). The three genes contain a cleaved signal peptide, predicting export of the protein in the periplasm or the extracellular medium. PUL 1 contains other gene-encoding enzymes predicted to be involved in alginate degradation: KgdF (PCAR9_a20872) which catalyzes the conversion of 4,5-unsaturated monouronates to 4-deoxy-L-erythro-5-hexoseulose acid (DEH, Hobbs et al., 2016); a sugar oxidoreductase (PCAR9_a20869) and a carbohydrate kinase (gene *KdgK*, PCAR9_a20874) whose homologs in *Z. galactanivorans* Dsij have been shown to convert DEH to 2-keto-3-deoxy-D-gluconate, and then to 2-keto-3-deoxy-6-phosphogluconate (KDPG) as final product (Thomas et al., 2012). KDPG can be further assimilated in the Entner-Doudoroff pathway (Preiss and Ashwell, 1962a,b). PUL 1 also encodes sugar transporters (an MFS transporter, PCAR9_a20873, and two TBDR, PCAR9_a20879, PCAR9_a20880) and a regulation factor gene (PCAR9_a20881). The two other alginate lyase genes are distant from PUL 1: a potential exo-alginate lyase belonging to the PL7_5 family (PCAR9_a20415) and predicted to be localized in the outer membrane as a lipoprotein and a PL18 family alginate lyase (PCAR9_a31210) appended to a carbohydrate-binding module of family 16. Therefore, *P. carrageenovora*’s genome contains all necessary genes involved in alginate catabolism and this was confirmed by bacterial growth on alginate as the sole carbon source (Table 2). Comparative genomics of PUL 1 indicate that the gene synteny is conserved in 19 *Pseudoalteromonas* genomes, belonging to the previously mentioned LDC in the phylogenetic tree (Supplementary Figure S1 and Supplementary Table S6). Comparison of PUL 1 to the genome of MHB from different lineages, namely [*P. atlantica*] T6c, *Zobellia galactanivorans* Dsij^T^, and *Flavobacteriaceae bacterium* S85 (Figure 4), indicated the presence of homologs encoding for either one or two of the polysaccharide lyases (PL6 family, gene *alyA6*, and PL17_2 family, gene *alyA3*), some transporting protein (MFS, TBDR) and some of the degradation proteins (*KdgF* and *KdgK*) in these genomes. The other two alginate lyases (PL7_5 family, PCAR9_a20415, and PL18 family, PCAR9_a31210) are localized elsewhere on chromosome I. Homologs of the PL7_5 and PL18 alginate lyases are found in 12 of the 19 genomes containing PUL 1. Other genomes contain either one or both genes and belong to the LDC in the phylogeny (Supplementary Figure S1 and Supplementary Tables S6, S7). Homologs of PCAR9_a20415 (PL7_5) are also found in [*P. atlantica*] T6c, *Z. galactanivorans* Dsij^T^ (gene *alyA5*) and *Flavobacteriaceae bacterium* S85 while no homologs of PCAR9_a31210 (PL18) were found in these 3 species.

#### Laminarin Catabolism

Beta-1,3-glucans include storage glucans such as laminarin in brown algae (Michel et al., 2010) and chrysolaminarin in diatoms (Myklestad, 1989), cell wall polymers of fungi and oomycetes (Aronson et al., 1967; Bartnicki-Garcia, 1968) and of brown algae (Raimundo et al., 2017). Both chromosomes of *P. carrageenovora* show putative activities on beta-1,3-glucans. Chromosome II contains a gene cluster (PUL 5) which includes a putative TBDR (PCAR9_b0364) and a GH16 endo-1,3-beta-glucanase (PCAR9_b0365) which could be active on very different types of biologically unrelated beta-1,3-glucans. Notably, PUL 5 is conserved in 47 of the 52 *Pseudoalteromonas* genomes suggesting an ancestral character of the genus. Chromosome I encodes two other GH16 endo-1,3-beta-glucanases (PCAR9_a21197 and PCAR9_a30350). Orthologs of these GH16 enzymes are only found in 2 species (*P. hodoensis* H7^T^ and *P.* sp. SM9913), suggesting recent horizontal acquisitions (Supplementary Figure S1 and Supplementary Tables S3, S6). Growth experiments have confirmed that this laminarin utilization system is functional (Table 2).

#### Starch Catabolism

Chromosome I contains a large gene cluster (PCAR9_a21164-PCAR9_a21179, PUL 2) predicted to be responsible for importing and utilizing extracellular starch, the storage polysaccharide of green and red algae (Supplementary Table S3). These predictions are consistent with the ability of *P. carrageenovora* to grow with starch as the sole carbon source (Table 2). Noticeably, PUL 2 is conserved in the 52 analyzed *Pseudoalteromonas* genomes.

#### Biosynthesis and Catabolism of Polysaccharides in the Biofilm

Bacteria associated with macroalgae may either be endo- or epiphytic (Egan et al., 2013). Additionally, *Pseudoalteromonas* species are known to efficiently produce biofilms (Bowman, 2007). Accordingly, the chromosomes of *P. carrageenovora* contain functions involved in exopolysaccharide biosynthesis and degradation in the biofilm. There is a bacterial cellulose synthase (*bcs*) operon on chromosome I (genes PCAR9_a20703-PCAR9_a20711), following the type IIa synteny: *bcsGFE*, *bcsRQABZC*, as defined by (Römling and Galperin, 2015). Cellulose is synthesized on the cytoplasmic side of the inner membrane and it results from the functional coupling of a transmembrane cellulose synthase (gene *bscA*, GT2 family) and a cyclic-di-GMP-binding protein (*bcsB*) located in the periplasm. Other genes of the operon are involved in enzymatic activity and product yield. It includes gene *bcsC*, encoding a periplasmic protein to guide cellulose outside of the cell; genes *bscZ* and *bcsE* which are involved in cellulose production, such as the packing of fibrils; gene *bcsQ*, encoding a protein with a role in cellular localization of the BCS complex; and gene *bcsR*, involved in biofilm formation (Supplementary Table S3). Twenty-six of the other *Pseudoalteromonas* genomes contain the corresponding type IIa synteny. In the 16S rRNA gene phylogeny, 19 *Pseudoalteromonas* strains of the LDC harbor the *bcs* operon in their genomes while the other 7 strains are distributed along the phylogeny (Supplementary Figure S1 and Supplementary Table S6). This suggests that this operon has been acquired in *Pseudoalteromonas* genomes from independent HGT events.

Sucrose is a disaccharide which can be used as substrate by bacteria to produce exopolysaccharides (Flemming and Wingender, 2010). Chromosome II contains two PULs involved in sucrose utilization, PUL 4: PCAR9_b0346-PCAR9_b0351 and PUL 6: PCAR9_b0546-PCAR9_b0549, which include the necessary genes for sucrose import, sucrose catabolism and transcription regulation (Supplementary Table S3). PULs 4 and 6 are present in a majority of *Pseudoalteromonas* genomes (32 and 27, respectively) and are distributed along the phylogenetic tree (Supplementary Figure S1 and Supplementary Table S6). Several bacterial species contribute to biofilm formation and structure maintenance by producing levan, an extracellular beta-2,6-polyfructan with extensive branching through beta-2,1-linkages (Laue et al., 2006). This EPS is synthesized from sucrose by an extracellular levansucrase (Osman et al., 1986). This enzyme is found on chromosome I (GH68 family, PCAR9_a30563), as well as a levanbiose-producing levanase (GH32 family, PCAR9_a30564). Therefore, *P. carrageenovora* likely produces extracellular levan as another structural polysaccharide of the biofilm. Homologs are present only in two *Pseudoalteromonas* strains, which are distant from *P. carrageenovora* in the phylogenetic tree (Supplementary Figure S1 and Supplementary Table S6), suggesting an acquisition from independent HGT events.

### Potential Virulence of *P. carrageenovora*

As expected, *P. carrageenovora*’s plasmid contains the genes of the replication/segregation system which is essential for plasmid maintenance (*rep* and *parA/B*, PCAR9_p0001-PCAR9_p0004, Friedman and Austin, 1988). It also encodes a gene cluster (PCAR9_p0005-PCAR9_p0014) potentially involved in a virulent character of *P. carrageenovora*. This cluster includes a two-component signal transduction system (a sensor histidine kinase *QseC*, PCAR9_p0008 and a DNA-binding response regulator *QseB*, PCAR9_p0009), a putative high affinity iron transporter gene (PCAR9_p0006), two long-chain acyl-CoA synthetase genes (PCAR9_p0012 and PCAR9_p0013) and a thermostable hemolysin gene (PCAR9_p0014). Iron transporters and two-component systems are known to play a role in the activation of virulence, as observed in the phytopathogen *Erwinia amylovora* (Zhao et al., 2009). The two long-chain acyl-CoA synthetase genes (PCAR9_p0012 and PCAR9_p0013) present in the cluster may regulate, for instance, cytosolic enzymes, ion channels, ion pumps, or genes (Faergeman and Knudsen, 1997). Hemolysin genes are also well-known bacterial virulence factors in several species of *Vibrio* involved in hemolysis of erythrocytes and in cytotoxicity (Nishibuchi and Kaper, 1995). When activated by iron, the *QseB*/*QseC* system of *P. carrageenovora* might be involved in the synthesis of the thermostable hemolysin. This gene cluster is conserved in 24 *Pseudoalteromonas* genomes distributed across the phylogenetic tree (Supplementary Figure S1 and Supplementary Table S7). Similar potential virulence clusters are also found in the genome of the pathogenic *Gammaproteobacteria Vibrio alginolyticus* 12G01, from which the thermostable hemolysin was characterized and shown to be toxic to zebrafish (Jia et al., 2010), and of the *Alphaproteobacteria Nautella* sp. R11, a red algal pathogen (Fernandes et al., 2011).

## Discussion

Marine bacteria of the *Pseudoalteromonas* genus are frequently found at the surface of macroalgae (Hollants et al., 2011; Martin et al., 2014). Some strains are capable of assimilating macroalgal polysaccharides (Goecke et al., 2010; Martin et al., 2015). Our genomic and physiological analyses demonstrate that this is the case of *P. carrageenovora*, which is able to feed on carrageenans and other macroalgal polysaccharides (starch, laminarin, alginate and fucoidans, Table 2 and Supplementary Table S4).

The most surprising outcome of our genomic study is the presence of a plasmid harboring all the carrageenan-related genes of the genome of *P. carrageenovora*. Plasmids usually represent a selective advantage for bacteria living under environmental pressure as they often carry antibiotic resistance genes or genes allowing the use of specific compounds such as novel carbohydrate sources. They are transmitted between bacteria through HGTs, which happen more often in conditions such as those in biofilms, representing places of high cell density and metabolic activity (Sørensen et al., 2005). Noticeably, plasmids are not ubiquitously present in genomes of the *Pseudoalteromonas* genus (Médigue et al., 2005; Qin et al., 2011). Carrageenan-specific genes are conserved in only six closely related *Pseudoalteromonas* strains (Supplementary Figure S1 and Supplementary Table S6), suggesting they also possess a similar carrageenan-related plasmid only recently acquired. This plasmid includes the genes encoding kappa and lambda family carrageenases but no 3,6-anhydro-D-galactosidases (GH127 and GH129 families, Ficko-Blean et al., 2017). Therefore, *P. carrageenovora* is able to damage red algal tissues containing kappa family carrageenans, but it is not able to assimilate alone the resulting products. Within a red algal epibacterial community, *P. carrageenovora* could grow with D-galactose and 3,6-anhydro-D-galactose released by other bacteria with a complete catabolic pathway for kappa family carrageenans, such as *Z. galactanivorans* Dsij (Ficko-Blean et al., 2017). Notably, none of the analyzed *Pseudoalteromonas* genomes display GH127 or GH129 genes, suggesting that these genes were already absent from the plasmid when it was acquired by *Pseudoalteromonas* strains. In contrast, the growth experiments demonstrate that the lambda-carrageenan catabolism is complete. Our comparative genomic analyses suggest that this catabolism is essentially conferred by PUL 8 and highlights the GH110 enzymes as strong candidates for the still unidentified lambda-carrageenan 1,3-alpha-D-galactosidase activity. Among the 52 *Pseudoalteromonas* genomes, the genome of *P. carrageenovora* is the only one possessing GH150 lambda-carrageenase genes and the PUL 8. The most parsimonious scenario is that these genes were not initially present on the ancestral plasmid and were subsequently acquired through another HGT event. Overall, this plasmid gives an ecological advantage to *P. carrageenovora* to use carrageenophyte red macroalgae as a source of nutrient. This edge might be increased by the additional presence of a potential cluster of virulence, as seen in some red algal pathogens (Fernandes et al., 2011).

The other carbohydrate metabolisms are encoded by chromosomes I and II. Even though they are localized on the main chromosomes, these pathways do not have the same evolutionary histories. Starch and laminarin catabolisms (PUL 2 and PUL 5, respectively) are conserved in almost all *Pseudoalteromonas* genomes, suggesting an ancestral acquisition in the genus (Supplementary Table S4). This ancient character is consistent with the prevalence of these polymers in coastal environments. Starch and laminarin are the storage polysaccharides of macroalgae (red/green algae and brown algae, respectively), and of abundant phytoplankton (starch: dinoflagellates; chrysolaminarin: diatoms, haptophytes). The ability to use these abundant polymers is an adaptive advantage for *Pseudoalteromonas* strains living in the water column as well as on the surface of algae. The majority of the *Pseudoalteromonas* strains (32/52 genomes) can degrade sucrose, but only three (including *P. carrageenovora*) can use the released fructose to synthesize the exopolysaccharide levan. Such EPS are important to build biofilm structures and *Pseudoalteromonas* species are known to play important roles in marine biofilms (Holmström and Kjelleberg, 1999; Bowman, 2007). Alginate catabolism in *P. carrageenovora* is essentially due to PUL 1 which is very similar to one alginate utilization system characterized in *Z. galactanivorans* Dsij. Thomas and coworkers provided phylogenetic evidence that alginate-specific PULs originated from marine flavobacteria and were independently transferred several times to marine *Gammaproteobacteria* (Thomas et al., 2012). Our comparative genomic analyses strengthen this hypothesis, since PUL 1 is only conserved in the *Pseudoalteromonas* strains of the late diverging clade (LDC; Supplementary Figure S1). Therefore, alginate catabolism is much older than carrageenan catabolism in the *Pseudoalteromonas* genus, but it nonetheless resulted from an ancient HGT event which most likely involved the common ancestor of the LDC. Considering its level of conservation within the LDC, alginate catabolism is surely a helpful trait for these strains to colonize the brown algal surface. Consistently, all the 22 *Pseudoalteromonas* strains isolated from the brown alga *Ascophyllum nodosum* by Martin and coworkers were alginolytic, whereas only a minority were carrageenolytic (Martin et al., 2015). Interestingly, most strains with the cellulose biosynthesis (*bcs*) operon also belong to the same LDC. For these strains, this suggests that the *bcs* operon was transferred alongside the alginate PUL during the same HGT event. Nonetheless, cellulose biosynthesis was acquired independently three other times in *Pseudoalteromonas* evolution (Supplementary Figure S1). Like levan, cellulose is secreted as part of the EPS matrix and has a structuring or surface attachment role in biofilms (Flemming and Wingender, 2010; Römling and Galperin, 2015). It also allows cell protection from hazardous effect of UV radiation (Scott Williams and Cannon, 1989). Therefore, cellulose biosynthesis can be considered as another trait for the adaptation to a life at the surface of macroalgae.

## Conclusion

In conclusion, the *Pseudoalteromonas* common ancestor likely had limited capacities to use complex carbohydrates, essentially focused on the storage polysaccharides of phytoplankton and macroalgae (laminarin and starch) which are abundant in coastal environments. Sucrose utilization is also a relatively ancient metabolic route in the *Pseudoalteromonas* genus. However, the adaptive traits genuinely specific for a macroalgae-associated lifestyle were acquired much later in *Pseudoalteromonas* evolutionary history through a succession of HGT events. A major event was the acquisition of alginate catabolism by the common ancestor of the LDC (likely from marine flavobacteria), a catabolism which is largely conserved in *Pseudoalteromonas* strains isolated from macroalgae. Probably the same HGT event has provided the capacity to synthesize cellulose, which helps to build biofilms on hosts and abiotic surfaces. In contrast, the ability to degrade kappa family carrageenan is more recent, due to a plasmid only found in 7 *Pseudoalteromonas* strains. Interestingly, these carrageenolytic strains are also found in the LDC. It seems that the acquisition of a first trait specific to macroalgae colonization (alginate catabolism) has favored subsequent HGTs. This could be explained by the close proximity between cells within a macroalgal biofilm, which facilitates genetic exchange (Hausner and Wuertz, 1999; Flemming and Wingender, 2010). Finally, *P. carrageenovora* is a particularly emblematic example of such successive acquisitions of adaptive traits since this species has even more recently acquired the pathway for the utilization of lambda-carrageenan.

## Author Contributions

AG, TB, and GuM designed the study. AG and TB carried out cultivation experiments and DNA extraction of the bacterium. GM performed the optical-mapping. ED and DV performed genome assembly, cleaning and automatic annotation with substantial intellectual contribution. AG performed genome annotation. AG, TB, MM-M, and GuM analyzed and interpreted the data. AG wrote the first draft of the manuscript and GuM contributed substantially to the revisions. All authors read and approved the final manuscript.

## Conflict of Interest Statement

The authors declare that the research was conducted in the absence of any commercial or financial relationships that could be construed as a potential conflict of interest.

## Footnotes

1 http://www.cazy.org

2 http://pfam.sanger.ac.uk

3 http://abims.sb-roscoff.fr/sulfatlas/
