# Supplementary material for: Evolutionary Evidence of Algal Polysaccharide Degradation Acquisition by Pseudoalteromonas carrageenovora 9T to Adapt to Macroalgal Niches
Source: Front Microbiol. 2018 Nov 22;9:2740. doi: 10.3389/fmicb.2018.02740 (PMC6262041; doi:10.3389/fmicb.2018.02740)
Supplement: Supplementary file 9 [file Data_Sheet_1.PDF]

Evolutionary evidence of algal polysaccharide degradation acquisition by *Pseudoalteromonas carrageenovora* 9^T to adapt to macroalgal niches

Angélique Gobet, Tristan Barbeyron, Maria Matard-Mann, Ghislaine Magdelenat, David Vallenet, Eric Duchaud and Gurvan Michel

Supplementary files

Supplementary figures:

Fig. S1. Phylogeny of the order *Alteromonadales* based on the 16S rRNA gene. Presence of the cellulose synthesis operon, the alginate operon and the carrageenolytic operon in genomes of the corresponding *Pseudoalteromonas* in the phylogenetic tree is indicated by a colored symbol: a green circle, an orange diamond and a red square, respectively (details in **table S6**). Only bootstrap values above 50% are shown.

Supplementary tables:

Table S1. List of all the carbon sources used to test growth of the bacterium *Pseudoalteromonas carrageenovora* 9^T.

Table S2. Number of annotated genes for each glycoside hydrolase, polysaccharide lyase and sulfatase family in the two chromosomes and the plasmid of *Pseudoalteromonas carrageenovora* 9^T. GH, Glycoside hydrolases; PL, Polysaccharide lyases. Sulfatase gene counts are indicated for each subfamily.

Table S3. Genes identified as putative CAZymes, sulfatases or other proteins involved in the metabolism of polysaccharides located in the chromosome I, the chromosome II and the plasmid. Colored boxes indicate the genes belonging to an operon or a PUL and the TonB-dependent receptors are indicated in bold. PUL, polysaccharide utilization loci.

Table S4. Details of the putative genes present in the genome of Pseudoalteromonas carrageenovora 9^T that may act to degrade the highlighted carbohydrate sources.

Table S5. All genes present on the plasmid and their putative functions. Colored boxes indicate the genes belonging to an operon or a PUL and the TonB-dependent receptors are indicated in bold. PUL, polysaccharide utilization loci.

Table S6. Screening of genes and gene clusters involved in carbohydrate degradation from the genome of *Pseudoalteromonas carrageenovora* 9^T found in other *Pseudoalteromonas* genomes that may have been acquired from HGT. Genes or cluster of genes are considered present when the identity between sequences reaches at least 30% and 80% of coverage. Presence of genes or gene clusters are indicated by 1, 0 otherwise. Presence of the cellulose synthesis operon, the alginate operon and the carrageenolytic operon is indicated by a colored symbol in the 16S rRNA gene phylogenetic tree (Fig. S1). PUL, polysaccharide utilization loci.

Table S7. Number of genes homologous to genes in the plasmid of *Pseudoalteromonas carrageenovora* 9^{T} present in 62 *Pseudoalteromonas* replicons corresponding to 52 *Pseudoalteromonas* genomes. Only genes with a sequence coverage of at least 80% and a seq. id. of 30% minimum were considered.

Table S8. List of the proteins corresponding to the gene clusters PCAR9_p0019-PCAR9_p0052 and PCAR9_p0067-PCAR9_p0073 on the plasmid of *Pseudoalteromonas carrageenovora* 9^{T} in the respective genomes of the *Gammaproteobacteria* [*Pseudoalteromonas atlantica*] T6c, the *Planctomycetes Rhodopirellula* sp. SWK7, and of the *Bacteroidetes Zobellia galactanivorans* DsijT and *Flavobacteriaceae* bacterium S85. Genes ID are indicated as stated in the MicroScope website. Their sequence identity with homologous genes in *P. carrageenovora* is indicated.
