# Supplementary figures and images for: Evolutionary Evidence of Algal Polysaccharide Degradation Acquisition by Pseudoalteromonas carrageenovora 9T to Adapt to Macroalgal Niches
Source: Front Microbiol. 2018 Nov 22;9:2740. doi: 10.3389/fmicb.2018.02740 (PMC6262041; doi:10.3389/fmicb.2018.02740)

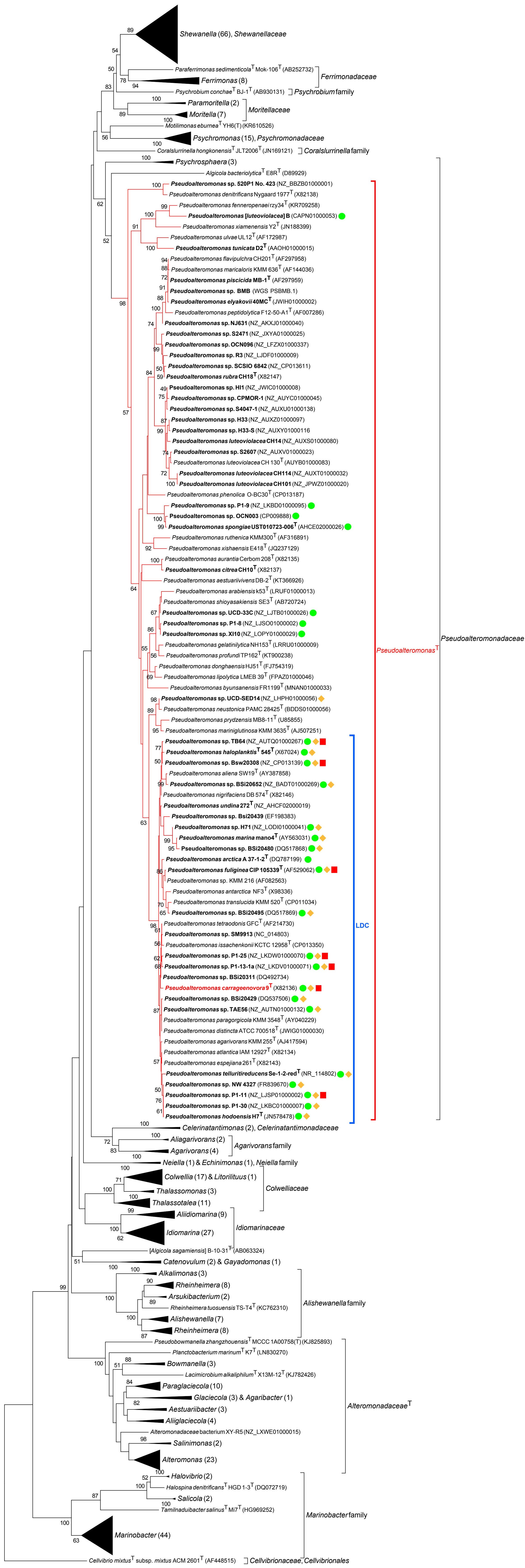

Supplement: Supplementary file 10 [file Image_1.PDF]
